# Evaluation of antibiotic consumption and resistance patterns among neonates: a 10-year retrospective study

**DOI:** 10.3389/fped.2026.1774875

**Published:** 2026-05-12

**Authors:** Wiona Denita Moras, Raushan Kumar Chaudhary, Soumya Patil, Seema Pavaman Sindgikar, Deepthi Avvaru, Uday Venkat Mateti

**Affiliations:** 1 Department of Pharmacy Practice, NGSM Institute of Pharmaceutical Sciences (NGSMIPS), Nitte (Deemed to be University), Mangalore, Karnataka, India; 2 Centre for Health Informatics and Evidence-based Medicine (CHIEM), Yenepoya (Deemed to be University), Mangalore, Karnataka, India; 3 Centre for Integrative Omics Data Science (CIODS), Yenepoya (Deemed to be University), Mangalore, Karnataka, India; 4 Department of Pharmacy Practice, Yenepoya Pharmacy College & Research Centre, Yenepoya (Deemed to be University), Mangalore, Karnataka, India; 5 Department of Pediatrics, KS Hegde Medical Academy (KSHEMA), Justice KS Hegde Charitable Hospital, Nitte (Deemed to be University), Mangalore, Karnataka, India; 6 Department of Infectious Diseases, Kasturba Medical College, Manipal Academy of Higher Education, Manipal, Karnataka, India

**Keywords:** antibiotics, days of therapy (DOT), defined daily dose (DDD), resistance, stewardship

## Abstract

**Background:**

Newborns are highly susceptible to infection, which leads to a higher chance of antibiotic utilization and resistance. Thus, the current study was carried out to evaluate antibiotic consumption, susceptibility, and resistance patterns among neonates.

**Methods:**

A retrospective study was conducted in a tertiary care hospital over 10 years to evaluate antibiotic consumption and resistance patterns among neonates. Antibiotic utilization was calculated using the defined daily dose (DDD) and days of therapy (DOT) based approach. All the data were analysed using SPSS version 29.

**Results:**

A total of 447 neonates were included in the study. Aminoglycosides (DDD:3089.449, DOT:2915), penicillin (DDD:3003.22, DOT:2218), and cephalosporins (DDD:1164.88, DOT:744) were the top three commonly prescribed classes of antibiotics. A total of 21 microorganisms were identified among neonates, of which *Coagulase-negative staphylococci* (17.88%), followed by *Klebsiella pneumoniae* (15.01%) and *Staphylococcus aureus* (12.14%)*,* were the top three prevalent microorganisms. Out of these 21 isolates, 16 were multidrug resistant (MDR), and 5 were non-MDR organisms. Aminoglycosides, penicillin, and lincomycin were the most resistant classes of antibiotics. Individually, gentamicin, followed by clindamycin, piperacillin/tazobactam, amoxicillin/clavulanic acid, meropenem, and cefoperazone/sulbactam were commonly resistant antibiotics. The estimated consumption of penicillin (DDD: *ρ* = 0.784, DOT: *ρ* = 0.76) and aminoglycosides (DDD: *ρ* = 0.745 and DOT: *ρ* = 0.758) significantly correlated with resistance patterns among neonates.

**Conclusion:**

Aminoglycosides and penicillin are the most commonly prescribed antibiotics with higher resistance among neonates, pointing towards the need for the implementation of an antimicrobial stewardship program in neonatal settings.

## Introduction

Antimicrobial resistance (AMR) poses a significant global health threat, with bacterial AMR directly responsible for 1.27 million deaths and associated with 4.95 million deaths worldwide in 2019 (1). In India, reports from the Indian Council of Medical Research (ICMR) indicate a worsening trend in AMR between 2016 and 2021 (2). Neonatal sepsis (NS) is a significant concern, particularly among very low birth weight (VLBW) infants, leading to increased complications and adverse neurodevelopmental outcomes (3). The widespread use of antibiotics in neonatal intensive care units (NICUs) has resulted in 74% – 94% of neonates with negative blood cultures receiving treatment for suspected sepsis (4). However, the indiscriminate use of empirical antibiotics poses a risk for the rise of AMR, which is linked to increased long-term morbidity and mortality, with 30% of NS deaths attributed to AMR (5). Higher consumption rates are associated with greater resistance to Gram-positive and Gram-negative bacteria. A Neo AMR network data from 39 neonatal units from 12 countries reported a 26% to 84% rate of resistance to at least one third- generation cephalosporin and 0% to 81% resistant rates to carbapenem against Gram-negative isolates, whereas from 0% to 45% resistant rates to glycopeptide against Gram-positive organisms (6). Similarly, various large-scale studies from worldwide conducted between 2011 and 2024 (NeoOBS, DENIS, BARNARDS, BabyGERMS, and CHAMPS) reported that the AMR rates for commonly used antibiotics vary between 42% to 69% for aminoglycosides, 59% to 84% for 3rd-generation cephalosporins, and 13% to 82% for carbapenems (7). Studies from low - and middle-income countries also show high resistance rates among Gram-negative bacteria, ranging from 26%–84% for cephalosporins and 0%–81% for carbapenems (8). Therapeutic management of neonatal infections is complicated by immunological immaturity and the limited number of antibiotics approved for neonatal use. Many antimicrobials are prescribed off-label due to a lack of neonatal-specific drug approvals (9). Neonates are also at increased risk of drug-related toxicity due to immature renal and hepatic function, altered protein binding, and rapidly changing body composition (10). Furthermore, randomised controlled trials in this population remain limited, and pharmacokinetic/pharmacodynamic (PK/PD) data are often extrapolated from older children or adults (11). As a result, dosing regimens are frequently based on limited evidence (12). Neonates are highly vulnerable to infection, with NS being a major concern. Thus, antibiotics are often used even when blood cultures are negative, increasing the risk of antibiotic resistance. Thus, it is necessary to evaluate the types of infection, antibiotic resistance, and consumption patterns among neonates. This highlights the pressing need to safeguard children's health through appropriate antibiotic stewardship (13). Thus, the current study was focused on antimicrobial stewardship (AMS) among the neonatal population.

Antimicrobial Stewardship (AMS) refers to the optimal selection, dosing, and duration of antimicrobial treatment to achieve the best clinical outcome while minimizing adverse effects and resistance (14). Various metrics are used to assess antibiotic use, including defined daily dose (DDD), days of therapy (DOT), length of therapy (LOT), and prescribed daily dose (PDD). However, DDD and DOT are both used together, as DDD quantifies the volume of antibiotic consumption, whereas DOT reflects the duration of therapy (15). However, DDD has limitations for certain antibiotics due to the lack of a standard correction factor, making the combined use of DDD and DOT more informative (16). Thus, the current study aimed to map the trends in antibiotic consumption and resistance in neonates over the duration of 10 years. Further, this study also correlated the antibiotic consumption with resistance patterns and compared the utility of DDD/DOT metrics in stewardship.

## Materials and methods

### Study site, sample size, and eligibility criteria

A retrospective study was conducted in the Department of Paediatrics at a tertiary care charitable hospital over 10 years, from January 2014 to December 2023. Ethical approval was obtained from the Institutional Ethics Committee (Ref. No: NGSMIPS/IEC/011/2024), and the study was registered with Clinical Trials Registry of India (Ref No: CTRI/2024/07//071126), ensuring privacy and confidentiality. Data on demographic details (age, weight, gender, date of admission, date of discharge), culture sensitivity test results, diagnosis, and antibiotic therapy were collected from the patients' case sheets in the medical records department. All the medical records of neonates admitted to the NICU and the neonatal wards were accessed to collect the data. The neonatal ward and the NICU are considered a single unit and are a dedicated separate department from the paediatric ward, with no mixed rooms. Nurses, members of the intensive care team, and specialized equipment are assigned exclusively to this unit and are not shared with other hospital wards. The hospital is a private institution with one dedicated neonatal unit that includes intensive care facilities. But the neonatal and NICU case files were stored electronically as a single unit under the International Classification of Diseases (ICD-10) using the code Z38.0 without segregating NICU vs. neonatal ward case files.

Neonates diagnosed with neonatal sepsis, pneumonia, meningitis, necrotizing enterocolitis, surgical site infection, osteomyelitis, septic arthritis, and umbilical sepsis based on clinical judgement, along with positive culture sensitivity (C&S) report, who received antibiotics for at least 5 days were included in the study. Antibiotic courses shorter than five days were excluded to ensure that only therapeutic regimens for confirmed infections were analyzed. In neonatal practice, shorter courses are often empirical, prophylactic, perioperative, or discontinued early due to negative culture reports. Including such brief exposures could overestimate antibiotic consumption and bias the resistance analysis, as these cases may not represent established infections. Thus, infection types were defined based on documented clinical diagnosis made by the treating neonatologist and supported by microbiological confirmation were included in this study. Antimicrobial susceptibility testing was performed using mainly the broth microdilution method (sometimes disk diffusion), and the result was interpreted according to the Clinical and Laboratory Standards Institute (CLSI) breakpoints. Any case sheets with unclear/incomplete information on demographic details, culture sensitivity, diagnosis, and treatment were also excluded from the study.

### Calculation of DDD and DOT

It is important to note that DDD is based on World Health Organization (WHO) adult standard dosing and does not account for age, weight, or gestation-specific dose adjustments required in neonates (17). In the present study, DDD was calculated specifically for the intravenously and orally (syrup) administered antimicrobials among neonates using the neonatal DDD proposed by Villanueva-Bueno et al. (16), instead of the WHO correction factor (Supplementary Table S1). DDD/100 bed days is the number of grams of antibiotics used   ×   100/(WHO DDD Units (g)  ×  Number of bed days (18). Similarly, days of antibiotic therapy (DOT) represent the days of antibiotic therapy administered to a patient regardless of the number of doses administered or dosage strength, whereas DOT/100 bed days is the sum of days of antibiotic therapy/Number of bed days (19). The formulas for DDD, DDD/100 bed days, DOT, and DOT/100 bed days are given below;

$$DDD=\frac{Total\;dose\;of\;drugs\;in\;grams}{Neonatal\;DDD\;(g)\;}$$

$$DDD/100\;bed\;days=\frac{No.\;\;of\;\;grams\;of\;antibiotics\;used\;\times \;100}{Neonatal\;DDD\;(g)\times \;No.\;of\;bed\;days}$$

No. of grams of antibiotic used = strength of unit dosage (g)  ×  No. of unit doses per package   ×  No. of packages used.

No. of bed-days = No. of beds in the hospital  ×  Occupancy index × No. of days (during the study period).

Occupancy index = Percentage of beds occupied during the study period.

DOT=SumofDaysofTherapyforparticluarAntibiotics

$$DOT/100\;bed\;days=\frac{Sum\;of\;days\;of\;\;Antibiotic\;Therapy\times 100}{No.\;of\;bed\;days\;}$$

### Calculation of the difference between DDD and DOT

A positive percentage difference indicates that the calculated antibiotic volume exceeds days of antibiotic therapy (DDD > DOT), which might reflect the inappropriateness of adult-based DDD calculation in neonates rather than true overdosing, if the WHO correction factor is used. However, we used the neonatal correction factor. Thus, the positive percentage difference reflects the high dose intensity, whereas the negative percentage denotes longer exposure at lower daily doses (DOT > DDD) in this study (19).

$$Difference\;between\;DDD\;and\;DOT\;=\frac{(DDD-DOT)\times 100}{DDD}\;$$

### Statistical analysis

The data was described using mean, standard deviations, frequency, and percentage. Further, the correlation between DDD, DD/100 bed days, DOT, and DOT/100 bed days, and between consumption pattern and resistance pattern was performed using Spearman's rank correlation. Similarly, the consumption value of individual antibiotics (DDD and DOT) was correlated with the number of patients resistant to particular antibiotics. All the statistical analyses were performed using SPSS software version 29. *P*-value <0.05 was considered significant.

## Results

### Study population and sample collection

Out of 16,860, a total of 447 neonates treated in the NICU and neonatal wards between the duration of 10-years (January 2014 and December 2023) were included in the study, whereas 206 records were excluded owing to incomplete information, and 16,207 were without culture sensitivity (C&S) report.

### Demographic details, clinical characteristics, and therapeutic regimen

Among the 447 included neonates, 48.54% were male, and 51.45% were female. The difference between male (51%) and female (49%) neonates was descriptive and not statistically significant. The overall median age was 5 days (3, 10). Most of the patients belonged to the age group ≤ 10 days (79.19%) with majority having a birth weight between 2 and 3 kg and length of hospital stay of 6 to 10 days whereas only 4.25% belonged to the age group of 21 to 30 days having a birth weight of > 4 kg with the length of hospital stay of 21 to 25 days. Respiratory-related infections are the most prevalent, accounting for 49.76%, followed by sepsis-related infections at 45.29%. Most of these infections are community-acquired compared to hospital-acquired. None of the neonates was diagnosed with surgical site infection or osteomyelitis. Regarding antibiotic therapy, the data showed that 68.00% of patients received monotherapy while 31.99% were treated with combination therapy, and none of the patients were treated with ambulatory antibiotic therapy (Table 1).

**Table 1:** Demographic details and clinical characteristics of neonates (*n* = 447) treated in NICU and neonatal wards.

Baseline characteristics	Frequency (n)	Percentage (%)
Sociodemographic details		
Gender		
Male	217	48.54%
Female	230	51.45%
Age (days)		
≤10 days	354	79.19%
11–20 days	74	16.55%
21–30 days	19	4.25%
Weight(g)		
<1 Kg	7	1.56%
1-<2 Kg	104	23.26%
2-<3 Kg	223	49.88%
3-<4 Kg	108	24.16%
>4 kg	5	1.11%
Length of hospital stay(days)		
5 days	14	3.13%
6–10 days	216	48.32%
11–15 days	116	25.95%
16–20 days	93	20.80%
21–25 days	8	1.78%
Diagnosis		
Sepsis-related infection	385	45.29%
Respiratory-related infection	423	49.76%
Intestinal disease	17	2.00%
UTI	16	1.88%
Meningitis	9	1.05%
Type of antibiotic therapy		
Monotherapy	304	68.00%
Combination therapy	143	31.99%

Sepsis-related infection includes early/late onset sepsis, septic arthritis, infection and sepsis, umbilical sepsis, suspected neonatal sepsis; Respiratory-related infection includes congenital pneumonia, ventilator-associated pneumonia, lower respiratory tract infection, respiratory distress syndrome; and Interstitial disease includes necrotizing enterocolitis. NICU, neonatal intensive care unit; UTI, urinary tract infection.

### Biological sampling and culture sensitivity assay

**Figure 1:**
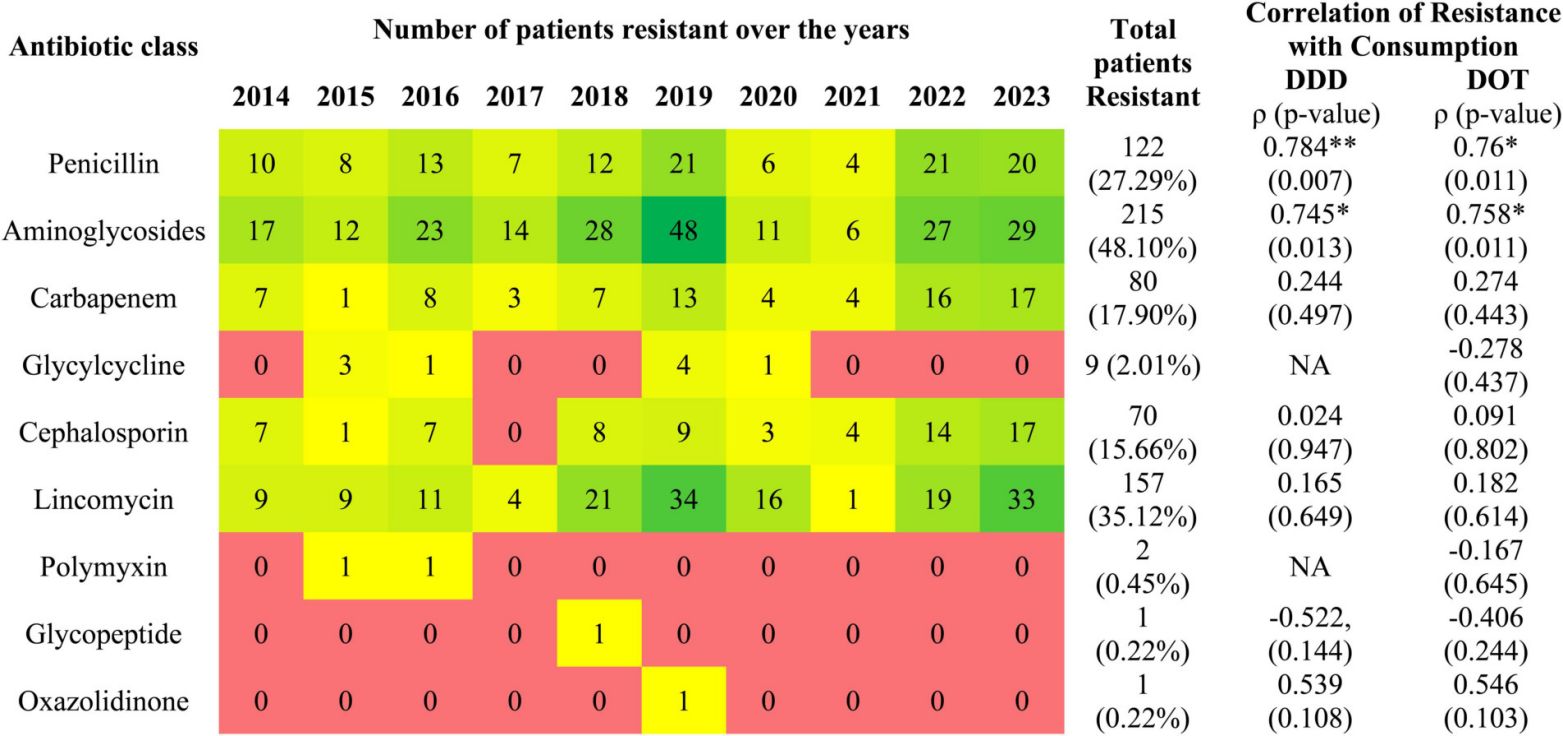
Heatmap showing yearly distribution of the number of neonates resistant to various classes of antibiotics and the correlation between consumption pattern and resistance pattern. None of the microorganisms isolated from neonates was resistant to nitroimidazole and macrolides. NA: Not applicable. *ρ* denotes the strength and direction of correlation, whereas * denotes the statistical significance *p*-value < 0.05 and ** denotes the statistical significance *p*-value < 0.01.

Only clinically indicated samples were included in the analysis, rather than surveillance cultures**.** In detecting organisms, liquid samples were commonly utilized, accounting for approximately 81.20%, followed by PUS (14.76%) and swabs (4.02%). All the neonates were infected with one organism, whereas six neonates were infected with two organisms each, making the total frequency 453. Out of 453, 269 infections (59.38%) were associated with Gram-positive organism whereas 184 infections (39.02%) were associated with Gram-negative organisms. A total of 21 microorganisms were identified among neonates, of which *Coagulase-Negative Staphylococci (CoNS*) (17.88%), followed by *Klebsiella pneumoniae (K. pneumoniae)* (15.01%) and *Staphylococcus aureus* (S. aureus*)* (12.14%)*,* were the top three commonly prevalent microorganisms (Table 2). Out of these 21 isolates, 16 were multidrug resistant (MDR), and 5 were non-MDR organisms. Based on the culture sensitivity (C&S) report, penicillin followed by fluoroquinolones, aminoglycosides, macrolides, lincomycin, cephalosporins, and sulfonamides were the most commonly resistant class of antibiotics to various micro-organisms. *CoNS* is resistant to penicillin (74 patients), macrolides (61 patients), fluoroquinolones (49 patients), and lincomycin (48 patients). Similarly, *K. pneumonia* is resistant to cephalosporin (61 patients), penicillin (60 patients), aminoglycosides (45 patients), and *S. aureus* is resistant to penicillin (54 patients), macrolides (52 patients), and lincomycin (47 patients) (Supplementary file Table S2). However, *CoNS* is highly sensitive to glycopeptide, oxazolidinone (77 patients), and tetracycline (69 patients). Similarly, S. *aureus* is sensitive to oxazolidinone (54 patients), glycopeptide (50 patients), sulfonamides (49 patients), *K. pneumoniae* is sensitive to sulfonamides (53 patients), and *MRCoNS* is sensitive to glycopeptide (46 patients), oxazolidinone (45 patients), and tetracycline (41 patients) (Supplementary file Table S3). In the current study, aminoglycosides (48.10%), followed by lincomycin (35.12%), and penicillin (27.29%), were the top three antibiotic classes resistant among neonates (Figure 1). Among the antibiotics, gentamicin (47.20%), clindamycin (35.12%), piperacillin/tazobactam (20.13%), amoxicillin/clavulanic acid (19.69%), meropenem (17.90%), and cefoperazone/sulbactam (15.44%) were the most commonly resistant antibiotics among neonates (Supplementary file Table S4).

**Table 2:** Biological sampling for culture sensitivity test, and classification of identified organisms.

A. Biological sample used for culture sensitivity test		
Culture specimen	Frequency (n)	Percentage (%)
**Swab**		
*Umbilical swab*	10	4.02%
*High vaginal swab*	8	
**Liquid sample**		
Blood	344	81.20%
CSF	6	
Urine	13	
**PUS**		
Umbilical tip	50	14.76%
ET tube tip	14	
Central venous tip	1	
Wound site	1	
B. Organism identified in culture sensitivity test		
Gram-positive Organism		
*Staphylococcus aureus*	55	12.14%
*Staphylococcus epidermidis*	5	1.10%
*Bacillus spp*	4	0.88%
*Enterococcus faecalis*	25	5.51%
*MRCoNS*	46	10.15%
*CoNS*	81	17.88%
*Gemella morbillorum*	2	0.40%
*Streptococcus agalactiae*	20	4.71%
*Alpha hemolytic streptococci*	2	0.44%
*Staphylococcus haemolyticus*	27	5.96%
*Staphylococcus hominis*	2	0.44%
Gram-negative Organism		
*Escherichia coli*	13	2.86%
*Pseudomonas aeruginosa*	18	3.97%
*Acinetobacter baumanii*	20	4.41%
*Klebsiella Pneumoniae*	68	15.01%
*Klebsiella oxytoca*	23	5.07%
*Acinetobacter Iwoffii*	6	1.32%
*Ralstonia picketti*	3	0.66%
*Enterobacter cloacae*	14	3.09%
*Enterobacter aerogenes*	7	1.43%
*Serratia marcescens*	12	2.64%

The frequency for biological samples denotes the number of patients, whereas the frequency for organisms detected denotes the number of times an organism was detected in neonates. All the neonates were infected with one organism except for 6 neonates, where they were infected with two organisms each, making the total frequency 453. CSF, cerebrospinal fluid; PUS, pus; ET, endotracheal; MRCoNS, methicillin resistant coagulase-negative staphylococci; CoNS, coagulase-negative staphylococci.

### Antibiotic consumption pattern using DDD and DOT

Initially, all the neonates were prescribed empirical antibiotic therapy based on clinical suspicion of infection, which was later modified based on the Culture and sensitivity report after 48–72 h. The prescription pattern was analyzed based on the number of times the antibiotics were prescribed among neonates. The most commonly prescribed antibiotics were aminoglycosides (555 prescriptions: 40.81%), followed by penicillin (432 prescriptions: 31.78%) and cephalosporin (139 prescriptions: 10.22%). Specifically, 6 antibiotics exhibited major differences (46%), 4 antibiotics showed moderate differences (30.7%), and 3 antibiotics had minor differences (23.07%). The antibiotics with the greatest differences included clindamycin and cefotaxime. At the same time, the smallest differences were noted for those typically administered once or twice daily with limited dosing ranges, such as syrup linezolid and piperacillin + tazobactam. The positive difference shows the increase in dosing intensity, whereas the negative difference shows the longer duration of therapy at lower doses (Table 3).

**Table 3:** Antibiotic therapy and comparison between DDD, DDD/100 bed days, DOT and DOT/100 bed days.

Antibiotics Drugs	Frequency	%	Total DDD	Total DOT	DDD/100 bed days	DOT/100 bed days	Difference between DDD and DOT (%)	Importance of the difference
**Penicillin**		31.78						
Ampicillin	290		2265.23	1514	130.54	82.32	33.16	Major
Piperacillin + Tazobactum	135		718.07	692	52.05	47.55	3.63	Minor
Syrup. Amoxicillin and Potassium Clavulanate	7		19.92	12	1.39	0.72	39.77	Major
**Aminoglycosides**		40.81						
Gentamicin	307		1756.649	1592	90.68	81.27	9.37	Moderate
Netilmicin	67		-	297	-	28.422	-	-
Amikacin	181		1332.8	1026	95.14	72.25	23.02	Moderate
**Carbapenem**		7.50						
Meropenem	102		747.86	717	46.38	45.88	4.13	Minor
**Glycylcycline**		0.44						
Tigecycline	6		-	13	-	0.723	-	-
**Nitroimidazole**		1.91						
Metronidazole	26		307.25	197	19.02	10.91	35.88	Major
**3rd generation cephalosporin**		10.22						
Cefotaxime	132		1164.88	692	83.12	50.32	40.60	Major
Cefoperazone and sulbactam	7		-	52	-	3.623	-	-
**Lincomycin**		0.14						
Clindamycin	2		8.65	5	0.53	0.25	42.20	Major
**Polymyxin**		0.07						
Colistin	1		-	7	-	0.24	-	-
**Glycopeptide**		5.22						
Vancomycin	71		320.59	395	21.45	23.99	−23.21	Moderate
**Oxazolidinones**								
Syrup. Linezolid	9	1.18	37.59	38	3.53	3.58	−1.09	Minor
linezolid	7		19.33	26	0.88	1.23	−34.47	Major
**Macrolides**		0.73						
Syrup. Azithromycin	10		12.28	13	0.52	0.55	−5.86	Moderate

Major (>25% difference), moderate (>5% and ≤25% difference), and minor (< 5% difference) importance. The % Difference between DDD and DOT calculated using the following expression: (DDD−DOT) X 100/DDD. The frequency and percentage are for the total number of times antibiotics prescribed, not the number of neonates. DDD, defined daily dose; DOT, duration of therapy.

The number of beds was 68 between 2014 and 2018, which was increased to 103 from 2019 onwards. Table 4 represents data on the number of beds, occupancy rates, and bed days. Occupancy rates fluctuated during this period, with a notable increase in 2022 when the rate for both DDD and DOT reached 0.079. Bed days started at 1,058 in 2014 for both DDD and DOT, decreasing to 860 in 2015 but seeing a significant rise from 2022, peaking at 2,963 and slightly declining to 2,922 in 2023. Table 5 outlines the antibiotic consumption measured in DDD per 100 bed days from 2014 to 2023 across various antibiotics. Ampicillin consumption peaked in 2023 with 509.21 DDD, while gentamicin showed a significant increase in 2023 at 380.63 DDD. Amikacin and meropenem also exhibited fluctuations, whereas vancomycin remained generally low. Usage patterns for less common antibiotics like metronidazole and different formulations like syrup azithromycin, and clindamycin were less pronounced, with many years reporting zero consumption. Table 6 shows antibiotic consumption measured in DOT per 100 bed-days from 2014 to 2023. Each year reflects varying levels of consumption for several antibiotics, such as ampicillin, gentamicin, cefotaxime, and others. Ampicillin showed a notable increase from 5.67 DOT in 2014 to 12.18 DOT in 2023, while gentamicin and meropenem exhibited fluctuating trends over the years. For instance, gentamicin rose significantly to 16.28 DOT in 2019 but decreased thereafter. Cefotaxime consumption varied, seeing a peak in 2016 at 12.15 DOT before declining. Other antibiotics like vancomycin and metronidazole demonstrated limited use, with a notable increase in specific years, signifying changes in treatment practices. Collectively, aminoglycosides (DDD: 3089.449 and DOT: 2915), followed by penicillin (DDD: 3003.22 and DOT: 2218) and cephalosporins (DDD: 1164.88 and DOT: 744), are the top three highly prescribed antibiotics among neonates.

**Table 4:** The number of beds, bed days, and occupancy rates of DDD and DOT from 2014 to 2023.

Years	2014		2015		2016		2017		2018		2019		2020		2021		2022		2023	
Consumption metrics	DDD	DOT	DDD	DOT	DDD	DOT	DDD	DOT	DDD	DOT	DDD	DOT	DDD	DOT	DDD	DOT	DDD	DOT	DDD	DOT
No. of beds	68	68	68	68	68	68	68	68	68	68	103	103	103	103	103	103	103	103	103	103
Occupancy rate	0.043	0.043	0.035	0.035	0.038	0.038	0.049	0.049	0.072	0.072	0.061	0.061	0.035	0.035	0.040	0.040	0.079	0.079	0.078	0.078
Bed days	1058	1058	860	860	946	946	1225	1225	1791	1791	2291	2291	1306	1306	1498	1498	2963	2963	2922	2922

**Table 5:** Yearly trends of antibiotics consumption among neonates based on DDD and DDD per 100 bed-days from 2014 to 2023.

Years	2014		2015		2016		2017		2018		2019		2020		2021		2022		2023	
Antibiotics	DDD	DDD/100	DDD	DDD/100	DDD	DDD/100	DDD	DDD/100	DDD	DDD/100	DDD	DDD/100	DDD	DDD/100	DDD	DDD/100	DDD	DDD/100	DDD	DDD/100
Ampicillin	88.78	8.39	252.89	29.40	44.54	4.70	183.72	14.99	261.57	14.60	437.06	19.07	98.80	7.56	38.10	2.54	350.53	11.83	509.21	17.43
Gentamicin	24.3	2.29	75.57	8.78	36.55	3.86	136.3	11.12	214.47	11.97	402.79	17.58	96.42	7.38	45.29	3.02	344.29	11.62	380.63	13.03
Cefotaxime	57.78	5.46	106.64	12.40	194.51	20.56	107.18	8.75	69.90	3.90	94.47	4.12	164.01	12.56	84.38	5.63	125.50	4.24	160.53	5.49
Amikacin	60.8	5.74	174.73	20.31	154.09	16.28	169.37	13.82	113.70	6.34	143.56	6.26	131.92	10.10	96.43	6.43	114.17	3.85	173.99	5.95
Meropenem	20.00	1.89	72.26	8.40	15.32	1.61	49.79	4.06	97.32	5.43	125.07	5.45	125.96	9.64	49.90	3.33	90.32	3.04	101.89	3.49
Piperacillin + Tazobactum	46.67	4.41	90.7	10.54	98.61	10.42	99.69	8.13	66.92	3.73	132.35	5.77	37.01	2.83	37.49	2.50	84.35	2.84	24.25	0.83
Vancomycin	23.25	2.19	54.77	6.36	26.16	2.76	11.53	0.94	10.43	0.58	53.15	2.32	18.11	1.38	21.41	1.42	43.38	1.46	58.38	2.00
Syrup. Linezolid	3.12	0.29	8.13	0.94	13.31	1.407	-	-	1.86	0.104	2.17	0.09	8.97	0.68	-	-	-	-	-	-
Metronidazole	-	-	37.8	4.39	55.2	5.83	12.70	1.03	-	-	54.06	2.36	-	-	11.12	0.74	44.16	1.490	92.19	3.155
Syrup. Azithromycin	-	-	-	-	1.1	0.11	-	-	0.68	0.03	1.37	0.06	-	-	-	-	2.16	0.07	6.96	0.24
Syrup. Amoxicillin + Potassium clavulanate	-	-	-	-	9.42	0.99	-	-	-	-	4.02	0.17	-	-	-	-	1.26	0.04	5.22	0.18
Clindamycin	-	-	-	-	-	-	-	-	-	-	-	-	5.62	0.43	-	-	3.01	0.102	-	-
linezolid	-	-	-	-	-	-	-	-	3.64	0.204	15.69	0.68	-	-	-	-	-	-	-	-

**Table 6:** Yearly trends of antibiotics consumption among neonates based on DOT and DOT per 100 bed-days from 2014 to 2023.

Years	2014		2015		2016		2017		2018		2019		2020		2021		2022		2023	
ANTIBIOTICS	DOT	DOT/100	DOT	DOT/100	DOT	DOT/100	DOT	DOT/100	DOT	DOT/100	DOT	DOT/100	DOT	DOT/100	DOT	DOT/100	DOT	DOT/100	DOT	DOT/100
Ampicillin	60	5.67	126	14.65	30	3.17	65	5.30	176	9.82	310	13.53	77	5.89	45	3.00	269	9.07	356	12.18
Gentamicin	55	5.19	52	6.04	33	3.48	90	7.34	201	11.22	373	16.28	78	5.97	48	3.20	316	10.66	346	11.84
Cefotaxime	73	6.90	64	7.44	115	12.15	60	4.89	34	1.89	39	1.70	79	6.04	46	3.07	58	1.95	124	4.24
Amikacin	97	9.16	120	13.95	98	10.35	104	8.49	98	5.47	117	5.10	98	7.50	67	4.47	94	3.17	133	4.55
Meropenem	39	3.68	78	9.07	18	1.90	45	3.67	68	3.79	130	5.67	107	8.19	61	4.07	86	2.90	85	2.90
Piperacillin + Tazobactum	65	6.14	71	8.25	63	6.66	88	7.18	70	3.90	143	6.24	33	2.52	38	2.53	94	3.17	27	0.92
Vancomycin	12	1.13	53	6.16	21	2.22	16	1.30	14	0.78	85	3.71	22	1.68	35	2.33	64	2.16	73	2.49
Syrup. Linezolid	9	0.85	9	1.04	9	0.95	-	-	2	0.11	2	0.08	7	0.53	-	-	-	-	-	-
Metronidazole	-	-	17	1.97	28	2.96	7	0.57	-	-	28	1.22	-	-	6	0.401	33	1.11	78	2.66
Syrup. Azithromycin	-	-	-	-	1	0.106	-	-	1	0.056	2	0.087	-	-	-	-	2	0.06	7	0.24
Syrup. Amoxicillin + Potassium clavulanate	-	-	-	-	4	0.42	-	-	-	-	3	0.13	-	-	-	-	1	0.03	4	0.13
Clindamycin	-	-	-	-	-	-	-	-	-	-	-	-	2	0.15	-	-	3	0.101	-	-
linezolid	-	-	-	-	-	-	-	-	8	0.44	18	0.78	-	-	-	-	-	-	-	-
Netilmicin	46	4.34	66	7.67	66	7.67	29	2.35	-	-	6	0.26	60	4.54	21	1.40	21	1.402	3	0.101
colistin	-	-	-	-	-	-	-	-	–	-	-	-	-	-	-	-	-	-	7	0.24
Tigecycline	-	-	-	-	-	-	-	-	7	0.39	-	-	3	0.23	-	-	1	0.03	2	0.06
Cefoperazone and sulbactam	-	-	7	0.814	7	0.814	-	-	-	-	15	0.65	7	0.536	8	0.534	7	0.236	1	0.03

**Figure 2:**
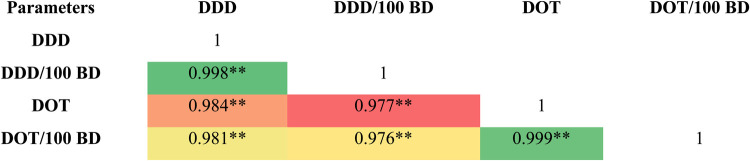
Heatmap for correlation between DDD, DDD/100 bed days (BD), DOT and DOT/100 bed days. The numbers inside the heatmap denotes the direction and strength of correlation whereas ** denotes the statistical significance *p*-value < 0.001. All the variables are positively and strongly correlated.

The correlation between DDD, DDD/100 bed days, DOT, and DOT/100 bed days was strong and significant (p < 0.001), indicating strong interrelationships among these metrics. Thus, both DDD and DOT can be used for analyzing the consumption of antibiotics. (Figure 2). Further, the yearly consumption pattern of penicillin (DDD: *ρ* = 0.784, *p*-value: 0.007 and DOT: *ρ* = 0.76, *p*-value: 0.011) and aminoglycosides (DDD: *ρ* = 0.745, *p*-value: 0.013 and DOT: *ρ* = 0.758, *p*-value: 0.011) estimated showed a significant correlation with their corresponding resistance pattern (Figure 1).

## Discussion

DDD is calculated as a proxy indicator using the adult WHO calculation factor (17). However, in the present study, we used the neonatal correction factor proposed by Villanueva-Bueno et al. (16), in which they calculated the neonatal DDD, which corresponds to the WHO correction factor. Further, DDD couldn't be calculated for various antibiotics due to a lack of a correction factor (Netilmicin, cefoperazone/sulbactam, tigecycline, and colistin), which eventually affects the total consumption of the whole class of drugs. Even though they are consumed by patients, it is not reflected by DDD. However, DOT mapped all the antibiotics. Thus, DOT is more actionable than DDD in neonates.

### Sociodemographic details and clinical characteristics of the neonates

Our study observed a higher prevalence of female neonates compared to males, which is parallel to the study conducted by Perera et al. (20), who reported female predominance (51.5%) over males (48.4%). This pattern was attributed to the presence of a well-established healthcare system with reduced gender bias and improved maternal care access. This study contrasts with the findings from secondary data analysis by Aghai et al. (21), where they found males (52.03%) were more predominant than females (47.97%)*.* Natural biological selection, differences in neonatal mortality rates, stillbirth rates, and cultural gender practices may influence this discrepancy. However, postnatal survival and healthcare access disparities may contribute to the variations in gender prevalence observed in different studies.

In terms of antibiotic prescribing patterns, monotherapy was the predominant approach observed among neonates in our study. This finding is comparable to the work of Sivanandan et al. (22), who conducted a randomized controlled trial evaluating the efficacy of amikacin and piperacillin + tazobactam as empirical monotherapies in neonates at risk of early-onset sepsis. Their study included a selected population of inborn neonates meeting specific gestational age and birth weight criteria, demonstrated no significant difference in treatment failure between the two monotherapy groups, thereby supporting single-agent empirical therapy in carefully defined settings. However, it is important to note that Korrang et al. (23), conducted a systematic review evaluating antibiotic regimens for late-onset neonatal sepsis, analyzed evidence from multiple randomized controlled trials, and concluded that current evidence is insufficient to establish the superiority of one antibiotic regimen over another, highlighting the need for individualized and protocol-guided antibiotic selection.

### Antibiotic therapy for neonates

In this study, aminoglycosides, particularly gentamicin, amikacin, and netilmicin, were primarily prescribed, followed by penicillin such as piperacillin + tazobactam, ampicillin, and amoxicillin + clavulanate which aligns with findings from a European study conducted by Metsvaht et al. (24), where they observed that penicillin constituted 36% and aminoglycosides constituted 30% of antibiotic prescriptions, with gentamicin being the most prevalent aminoglycoside at 78%. In contrast, an Indian study by Pushpa et al. (25), identified piperacillin + tazobactam as the most frequently prescribed antibiotic at 35.4%, with gentamicin and amikacin following at 30.2% and 12.29%, respectively.

### Most common microorganisms isolated

In the present study, *K. pneumoniae* (15.36%) and *CoNS* (17.62%) emerged as the predominant Gram-negative and Gram-positive organisms respectively, which is parallel to a south Indian study conducted by Karimi et al. (18), where *K. pneumoniae* (22.4%) and *CoNS* (21.9%) were reported to be most frequently identified pathogens since the organisms mentioned above are opportunistic pathogens that thrive in NICU and hospital settings. Both studies are due to NICU environments, neonatal immune vulnerabilities, and antibiotic resistance trends. In contrast, a study by Bizzarro et al. (26), at Yale-New Haven Hospital identified *Escherichia coli* (*E. Coli*) as the leading pathogen in early-onset sepsis, accounting for 45% of cases, followed by Group B *Streptococcus* (GBS) at 36%. These pathogens are more commonly associated with community-acquired infections and are particularly prominent in early-onset cases.

### Resistance/sensitivity pattern of the microorganism

In the present study, *CoNS* showed high resistance to penicillin, macrolides, fluoroquinolones, and lincomycin. Similarly, *K. pneumonia* demonstrated significant resistance to penicillin, cephalosporin, and aminoglycosides, while *S. aureus* was highly resistant to penicillin, macrolides, and lincomycin, indicating a concerning level of MDR among Gram-positive and Gram-negative neonatal pathogens. Similarly, a systematic review conducted by Anwer et al. (27), reported high resistance among *CoNS* to macrolides and beta-lactam antibiotics, and significant resistance among Klebsiella species to ampicillin and cephalosporins. These findings support the increasing prevalence of AMR among both Gram-positive and Gram-negative pathogens in neonatal intensive care units. In contrast, a study by Yeshiwas et al. (28), published in antimicrobial resistance and infection control reported lower resistance rates to aminoglycosides, particularly amikacin (3.0%–5.56%), suggesting retained effectiveness against *K. pneumoniae*. These findings differ from the present study, where *K. pneumoniae* showed high resistance to aminoglycosides. This variation may be attributed to differences in regional antibiotic prescribing practices, AMS policies, and infection control measures across neonatal intensive care units**.** However, *CoNS* demonstrated high sensitivity to glycopeptides, oxazolidinones, and tetracyclines. Similarly, *S. aureus* was highly sensitive to oxazolidinone, glycopeptide, and sulfonamides, while *K. pneumoniae* showed good sensitivity to sulfonamides. *MRCoNS* also retained marked sensitivity to glycopeptides, oxazolidinones, and tetracycline. Our findings are consistent with the study conducted by Song et al. (29), which reported 100% sensitivity of *CoNS* and *S. aureus* to vancomycin over a 20 - year period in NICU. This highlights the continued effectiveness of glycopeptides against Gram-positive neonatal sepsis pathogens. In contrast, a study by Deress T et al. (30), from the University of Gondar Comprehensive Specialized Hospital, Ethiopia, reported alarming resistance patterns among Gram-positive pathogens, including *CoNS* and *S. aureus,* where *CoNS* exhibited high resistance to multiple antibiotic classes and, notably, a subset of Gram-positive isolates showed reduced susceptibility to vancomycin. Although complete resistance was not universal, the emergence of vancomycin-resistant or less susceptible strains signals a concerning shift in antimicrobial susceptibility patterns.

In the present study, Gram-positive pathogens were more predominant than Gram-negative pathogens. This finding is consistent with the systematic review conducted by Fleischmann-Struzek et al. (31), which reported a higher proportion of Gram-positive organisms in several high-income settings, particularly *CoNS*, especially in late-onset sepsis in the NICU setting. In contrast, Folgori et al. (32), reported that Gram-negative organisms such as *K. pneumoniae* and *Acinetobacter baumannii* frequently predominate in many low and middle-income countries, particularly in parts of Asia and Africa. This predominance is often attributed to higher environmental colonization pressure, infection control challenge, and widespread empirical broad-spectrum antibiotic use. These regional differences likely contribute to variations in perinatal care practices, antimicrobial stewardship implementation, availability of microbiological diagnostics, and local resistance ecology. Therefore, comparisons across studies should consider differences in healthcare infrastructure, surveillance systems, and socioeconomic context when interpreting variability in pathogen distribution**.**

### Comparison between DDD, DDD/100 bed days, DOT, and DOT/100 bed days

Ampicillin was found be highly prescribed antibiotics based on its DDD and DDD/100 bed days which is similar to an Indian study by Jinka et al. (33), who observed higher utilization of ampicillin (∼5.68–5.8 DDD/100 patient-days) and substantial use of gentamicin (∼3.01–3.02 DDD/100 patient-days), whereas third-generation cephalosporins (cefotaxime) had lower DDD values and showed a significant reduction following the implementation of an antibiotic stewardship policy. Similarly, a study conducted by Karimi et al. (18), reported the highest cefotaxime utilization of 8.267 DDD/ 100 bed days among neonates, which is nearly parallel to our finding, where we observed cefotaxime to be the fourth most commonly prescribed antibiotic (83.12 DDD/100 bed days). This differs from the pattern observed in our study, suggesting potential variations in prescribing practice, local antimicrobial resistance patterns, and institutional AMS policies. In contrast, a Spanish study by Valles et al. (19), found that meropenem had the highest DDD, DOT, DDD/100, and DOT/100 bed days, followed by piperacillin/tazobactam and amoxicillin/clavulanic acid. The differences between DDD and DOT showed major differences for meropenem, while the latter two combination drugs showed major and moderate differences.

The dose size changes with the weight, and dosing frequency often changes with the gestational/post-natal age (34). DDD represents the aggregate dose given on a particular day, whereas DOT counts a day regardless of frequency/dose size (15). The significant correlation between DDD and DOT in our neonatal cohort denotes that the variability in antibiotic consumption is primarily driven by the therapy duration instead of differences in dose intensity. The DDD in neonates is often influenced by dose size and dosing frequency, despite the use of a neonatal correction factor. Even though the neonatal correction factor was used in this study, it cannot fully capture the gestational age-specific pharmacokinetic variability. Thus, DDD and DOT are not interchangeable but complementary to each other. However, DOT is more informative and appropriate than the DDD among the neonatal population (34).

### Impact of COVID-19 on antibiotic consumption among neonates

The study period (2014–2023) included the COVID-19 pandemic years (2020–2021), which influenced antibiotic utilization patterns. A decline in total DDD was observed in 2020 compared to 2019, along with a reduction in DOT for most antibiotics, except cefotaxime. In 2021, DDD and DOT remained lower than pre-pandemic levels, although relative increases were noted for vancomycin and piperacillin/tazobactam. This overall reduction in antibiotic use may be attributed to restricted healthcare access, reduced hospital admissions, and altered admission patterns. Additionally, disruptions in antimicrobial stewardship activities and shifting clinical priorities may have contributed to these changes (35).

### Limitations and future direction of the study

Although the current study involves an appropriate number of neonatal data for analyzing antibiotic resistance and consumption patterns among neonates, the study has several limitations. Firstly, its retrospective design and single-centre setting may limit the generalizability of the findings. The inclusion of only cases with available culture and sensitivity reports may have introduced selection bias. The study also did not evaluate the impact of antibiotic therapy on clinical outcomes such as length of hospital stay, clinical cure, or mortality among neonates. Furthermore, the retrospective nature of the study precluded real-time assessment of prescribing practices and stewardship interventions. The electronic medical records were stored as a single unit under the International Classification of Diseases (ICD-10) using the code Z38.0 without segregating NICU vs. neonatal ward case files, which didn't permit us to analyze their consumption pattern separately. Further, the gestational age of neonates was not collected during this study. Thus, future work can be focused towards a prospective longitudinal or ecological, multicentric study with structured time-series, along with consumption (DDD and DOT) and resistance pattern, which will provide real-time monitoring, a broad range of data collection (gestational age, NICU vs. neonatal ward), and stronger causal inferences regarding antibiotic consumption and resistance patterns.

## Conclusion

This study concludes that ampicillin, gentamicin, amikacin, and cefotaxime are the most widely prescribed antibiotics among neonates, of which gentamicin is highly resistant among neonates, while others remain viable. Thus, ampicillin, amikacin, and cefotaxime can be considered for empirical therapy among neonates. The prescription of antibiotics should preferably be based on the C&S test. However, broad-spectrum antibiotics with high sensitivity/low resistance should be considered for empirical use, while the antibiotics with a high risk of resistance should be considered only after the C&S report. Antibiotic stewardship program (ASP) should be implemented in neonatal settings to promote the strict guideline adherence for rational prescription and to minimize overuse of broad-spectrum antibiotics, which will ultimately counteract the surge of resistance. Prospective audit and feedback, preauthorization, and de-escalation should be practised in order to promote the judicious use of antibiotics, thereby enhancing clinical cure and decreasing the length of stay and mortality.

## Data Availability

The original contributions presented in the study are included in the article and Supplementary Material, further inquiries can be directed to the corresponding author.
